# State of Diabetes Self-Management Education in the European Union Member States and Non-EU Countries: The Diabetes Literacy Project

**DOI:** 10.1155/2018/1467171

**Published:** 2018-04-17

**Authors:** Henna Riemenschneider, Sarama Saha, Stephan van den Broucke, Helle Terkildsen Maindal, Gerardine Doyle, Diane Levin-Zamir, Ingrid Muller, Kristin Ganahl, Kristine Sørensen, Peter Chang, Dean Schillinger, Peter E. H. Schwarz, Gabriele Müller

**Affiliations:** ^1^Medical Clinic 3, University Hospital Carl Gustav Carus, Technische Universität Dresden, Dresden, Germany; ^2^Institut de Recherche en Sciences Psychologiques, Université Catholique de Louvain, Louvain-la-Neuve, Belgium; ^3^Department of Public Health, Aarhus University, Aarhus, Denmark; ^4^College of Business, University College Dublin, Dublin, Ireland; ^5^Clalit Health Services, Tel Aviv, Israel; ^6^Department of Psychology, University of Southampton, Southampton, UK; ^7^Gesundheit Österreich GmbH (Austrian Public Health Institute), Vienna, Austria; ^8^Department of International Health, Maastricht University, Maastricht, Netherlands; ^9^Ministry of Health & Welfare, National Taipei Hospital, New Taipei City, Taiwan; ^10^Center for Vulnerable Populations, Division of General Internal Medicine, University of California, San Francisco, CA, USA; ^11^Center for Evidence-based Healthcare, Medical Faculty, University Hospital Carl Gustav Carus, Technische Universität Dresden, Dresden, Germany

## Abstract

**Background:**

Diabetes self-management education (DSME) is considered essential for improving the prevention and care of diabetes through empowering patients to increase agency in their own health and care processes. However, existing evidence regarding DSME in the EU Member States (EU MS) is insufficient to develop an EU-wide strategy.

**Objectives:**

This study presents the state of DSME in the 28 EU MS and contrasts it with 3 non-EU countries with comparable Human Development Index score: Israel, Taiwan, and the USA (ITU). Because type 2 diabetes mellitus (T2DM) disproportionately affects minority and low-income groups, we paid particular attention to health literacy aspects of DSME for vulnerable populations.

**Methods:**

Data from multiple stakeholders involved in diabetes care were collected from Feb 2014 to Jan 2015 using an online Diabetes Literacy Survey (DLS). Of the 379 respondents (249 from EU MS and 130 from ITU), most were people with diabetes (33% in the EU MS, 15% in ITU) and care providers (47% and 72%). These data were supplemented by an expert survey (ES) administered to 30 key informants.

**Results:**

Access to DSME varies greatly in the EU MS: an average of 29% (range 21% to 50%) of respondents report DSME programs are tailored for people with limited literacy, educational attainment, and language skills versus 63% in ITU. More than half of adult T2DM patients and children/adolescents participate in DSME in EU MS; in ITU, participation of T1DM patients and older people is lower. Prioritization of DSME (6.1 ± 2.8 out of 10) and the level of satisfaction with the current state of DSME (5.0 ± 2.4 out of 10) in the EU MS were comparable with ITU.

**Conclusion:**

Variation in availability and organization of DSME in the EU MS presents a clear rationale for developing an EU-wide diabetes strategy to improve treatment and care for people with diabetes.

## 1. Introduction

As the number of people with diabetes mellitus (DM) steadily increases worldwide, health systems are obliged to consider cost-effective measures to fight the growing burden of diabetes and to improve health and life quality of people with diabetes. Several initiatives and position papers call for actions to improve the prevention as well as the quality of treatment and care of diabetes [1–6]. These studies mention the self-management capacity of patients as one of the critical success factors for addressing diabetes and improving treatment outcomes. This capacity, which is associated with the level of health literacy, can be improved through diabetes self-management education (DSME) [7–10]. DSME stands for an ongoing, individualized, multifaceted process to support patients to integrate self-management into their daily lives by improving knowledge and skills. It aims at improving quality of life and reducing subsequent complications by enabling informed decision-making for self-directed behavior changes and problem solving by active collaboration with health care team [11].

To date, there is little information regarding the state of DSME at the EU level. According to the Global Diabetes Scorecard [12], which monitors national governments' responses to the diabetes epidemic, 18 out of 28 European Union Member States (EU MS) have thus far introduced national diabetes plans or policy frameworks and to date only partially implemented. Nevertheless, a recent review of DSME arrangements in six European countries reported a policy shift towards more patient-centered self-management of diabetes in the primary care context [13]. The review highlights that strengthening the role of diabetes specialist nurses, multidisciplinary approaches, and a focus on patients are essential for the success of such programs. In addition, the relevance of socioeconomic circumstances for self-management capacity must be acknowledged. Beyond the national level strategies and policies, more information regarding the organization, structure, content, and cost-effectiveness of existing DSME programs in the community settings is needed for developing a comprehensive plan to fight diabetes in the EU.

As the capacity of patients to self-manage their disease is associated with their level of health literacy, more information is also needed regarding the role of health literacy in DSME. Data on the health literacy of the European population were collected via the European Health Literacy Survey (HLS-EU) [14], showing that 12% of the study population had insufficient health literacy and almost one in two Europeans (47%) had limited health literacy. The HLS-EU also demonstrated an association between limited health literacy, high demand for health services, and poor health status. It has been documented that when DSME takes poor health literacy into account by using literacy-appropriate educational materials and brief counseling in primary care settings, health outcomes of people with limited (health) literacy improve [7, 15–17]; as well it can also benefit people with higher levels of literacy. While addressing low health literacy is becoming increasingly recognized as a public health goal and a determinant of health outcomes [18], there is little evidence regarding how literacy is included in DSME in the EU MS.

This study aims at bringing new evidence regarding the state of DSME across the 28 EU MS, with a special focus on vulnerable populations such as children, elderly, and people with low health literacy levels, with a view to develop evidence-based recommendations to improve the effectiveness of DSME. The study was part of the Diabetes Literacy (DL) project [17] (http://www.diabetesliteracy.eu), which ran from 2012 to 2015 with support from the European Commission. It involved a novel approach that combined information from published and unpublished data related to DSME policies and programs of multiple stakeholders representing different diabetes-related groups (patients with diabetes, their relatives, care providers (i.e., general practitioners, diabetes nurses), diabetes-related experts, health insurance, and scientists).

## 2. Methods

### 2.1. Design

In order to map the state of diabetes self-management education (DSME) in the 28 EU MS, a database of DSME programs was compiled, and the perspectives of diabetes-related stakeholder groups (patients, care providers, experts, and other stakeholders) were analyzed with regard to organization, target groups, contents, delivery type, and role of DSME in the EU MS. Next, the EU data were compared and contrasted with three non-European project partner countries (Israel, Taiwan, and the USA (ITU)) presenting different health systems and cultures that have a comparably high Human Development Index score. This score is a method that allows comparison of average achievement in key dimensions of human development: a long and healthy life, being knowledgeable and having a decent standard of living [19].

### 2.2. Instruments and Data Collection

The comparative analysis was based on survey data and a literature review on national DSME strategies and programs in the EU MS [20]. The development of the survey tool involved a series of steps. Firstly, all relevant items describing current diabetes self-management education initiatives were collected [11, 21–29]. The items were categorized according to the group of stakeholders they targeted, to establish three separate instruments targeting different stakeholders. These instruments were then developed further consensually by a multiple Delphi process carried out within the project consortium. The two resulting instruments were used to collect data for this study from a diversity of stakeholders regarding DSME:

1. An online survey questionnaire, the Diabetes Literacy Survey (DLS), was developed to assess experiences and insights of patients, caregivers, and other stakeholders with regard to the state of DSME in their region. DSME programs were collected with an additional wiki tool (for detailed description, please refer to [30]). The DLS was translated into English, German, Spanish, French, Dutch, Hebrew, and Mandarin and made available online in partnership with the Global Diabetes Survey (GDS) [31] from February 2014. The GDS (http://www.globaldiabetessurvey.com) is an ongoing initiative to collect data on diabetes care quality on a yearly basis carried out on the initiative of TU Dresden. Recruitment to register and complete the DLS was carried out by inviting previous GDS members and the networks of the project consortium members through online networking, personal contacts, and public calls. The survey was targeted at people directly or indirectly involved in the care of people with diabetes, diabetes patients and their families, health professionals, and researchers as well as patient organizations. The survey remained accessible for a 12-month period (until the end of January 2015), after which the data were anonymized for the analysis.
2. An expert survey (ES) was developed to obtain information from experts on the health policy and economic aspects regarding DSME in the participating countries. Key informants in the field of patient education in the EU, Israel, and Taiwan (patient/diabetes organizations, scientific societies, large health insurance companies, health ministries, and education organizations/associations) were selected by reviewing DSME relevant websites and publications as well as by drawing on the existing networks of the consortium. Data collection began in May 2014 by inviting the identified stakeholders individually by email and/or phone calls to take part in the survey. The expert perspectives on DSME were used to verify the data collected through DLS and to obtain supraregional views on the DSME.

### 2.3. Ethical Approval

The study was conducted according to the Helsinki Declaration. Ethical approvals were obtained from the Ethical Committee, Medical Faculty, Technische Universität Dresden, as well as from all other respective project partners.

### 2.4. Analysis

An exploratory and descriptive analysis was made of the responses obtained in 2014 to the DLS and ES from multiple diabetes-related stakeholders (e.g., patients, family members, relatives, care providers, and experts). Group comparisons were done by chi^2^ test for responses related to categorical variables and by *t*-test for numeric variables. Country-specific analysis was restricted to variables for which a minimum of 8 participants had replied for each country, to assure the reliability of the results. In the event of fewer than 8 respondents per country, the results were included in the total EU sample and not analyzed individually. The EU data were also compared and contrasted qualitatively with Israel, Taiwan, and the USA.

## 3. Results

The survey was completed by 379 respondents (249 from EU MS and 130 from Israel, Taiwan, or the US (ITU)). The response varied significantly across the 31 participating countries, ranging from 1 to 70 participants in the EU MS and 9 to 95 in the other selected countries. EU MS with 8 or more participants (Austria, Belgium, Denmark, Germany, Ireland, Portugal, Romania, Spain, and the United Kingdom) were included in the country-specific analysis. Out of the total EU MS sample, 33% of the respondents were people personally affected by diabetes (patients, family members, and relatives), 47% were care providers (i.e., diabetes specialists, nurses, and physicians), and 20% “others” (i.e., policy makers, researchers, and health insurance). In the non-EU countries, these rates are 15%, 72%, and 12%, respectively.

The patient and provider data derived from the DLS were supplemented by responses on the expert survey (ES) by 23 diabetes experts from the EU and 7 from outside the EU (1 from France, Luxemburg, Poland, Portugal, Slovenia, and the UK; 2 from Belgium, the Czech Republic, Ireland, and Spain; 3 from Austria, Germany, Lithuania, and Taiwan; and 4 from Israel).

### 3.1. Landscape of DSME Programs

**Figure 1:**
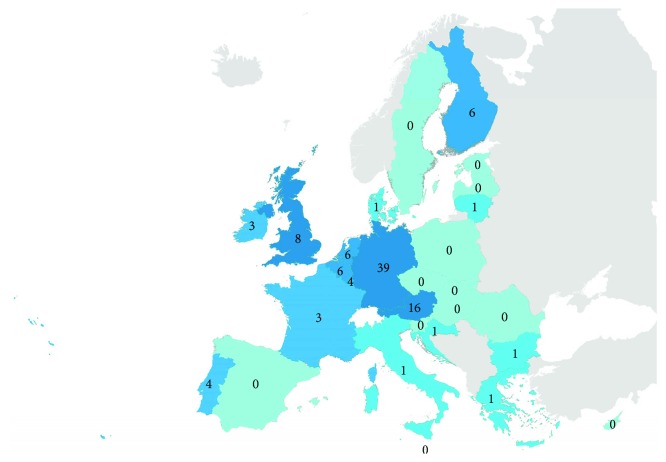
Number of DSME programs in the EU MS, based on Diabetes Literacy Survey-wiki (reported on http://dsme.wiki/ [20, 30]).

A total of 102 DSME programs were reported in DLS-wiki for the EU MS. The majority (76%) of the programs were targeted at adults, 8% for children and 6% for elderly people. The availability of programs varied significantly between countries, ranging between 0 and 39 programs (Figure 1). In Eastern and Southern Europe, in particular, fewer programs were reported. For Israel and Taiwan, 6 programs were identified, for the USA 25.

### 3.2. Providers of DSME

Respondents within the EU MS reported that DSME is most commonly provided by diabetes specialists (diabetologist/endocrinologist, 61%), followed by diabetes/health educators (50%), diabetes nurses (47%), and general practitioners (45%). Other providers are health insurance providers (9%) or other health-related organizations (7%) (multiple choice was possible) (Table 1). Interestingly, the answers regarding the providers of programs varied depending on the respondent group: care providers more often mentioned diabetes educators and primary care nurses as providers compared to the affected people (patients, family members, and relatives) (47% versus 25% and 27% versus 7%, resp.). There were also large variations between EU MS: for Germany, 91% of the respondents mentioned diabetes educators, while for Romania and Spain, these were not at all mentioned; in Portugal, 67% mentioned primary care nurses as DSME providers, whereas in Austria, Belgium, Ireland, and Romania, these were not mentioned. The largest variation was reported regarding self-directed learning: of the affected people in the EU, 31% mentioned this form of education, as opposed to 11% of the care provider. In comparison, in non-EU countries, 52% of the affected people and 32% of the educators mentioned self-directed learning.

Of the total EU MS sample, 45% of respondents reported that attending physicians actively provide self-management support to their patients directly “sometimes or occasionally,” 31% “often,” 8% reported “never, but referred to another healthcare professional,” and only 4% (range 0%–50% in different EU MS) responded “never, education not offered.” 10% of the respondents answered “don't know.”

### 3.3. Target Groups of DSME

Seventy-three percent (73%) of the respondents in EU MS reported that structured DSME programs exist in their community for adults with type 2 and type 1 diabetes mellitus (T1DM, T2DM), but there is a large difference between MS (92% in Ireland for T2DM programs versus 50% for Spain). In contrast, only 42% of the respondents reported the existence of DSME programs targeting older people, again with differences between countries (15% in Ireland and 60% in Germany). Of the total group of respondents from the EU, 61% mentioned the existence of DSME programs for children and 33% for peers and relatives of people with diabetes (Table 2).

Compared to care providers, affected people less often reported programs for children (52/66%), peers/relatives (28/42%), older people (33/49%), adults with T1DM (62/82%) or T2DM (58/84%), and women with gestational diabetes (27/58%). However, these differences regarding the responses from affected people versus care providers were not statistically significant (chi^2^ test: *p* = 0.676). Care providers appeared to have a better overview of the existing programs compared to the affected people (12% versus 34% “don't know”). The respondents in the non-EU countries reported similar or somewhat better estimates than those of the EU MS.

### 3.4. Literacy Aspects in DSME Design

Of the DLS respondents in the EU, 29% confirmed that there are DSME programs that are accessible for people with limited literacy, educational attainment, and language skills, with percentages ranging between 21% in Portugal and 50% in Denmark. Of the non-EU countries, 63% of the respondents considered DSME programs to be accessible for people with limited literacy (*p* < 0.001). When looking at the respondent groups, of the affected people, a majority (61% EU/58% other) of the respondents did not answer this question (Table 3). Of the care providers in EU MS, 44% were aware of such programs (range 13% Austria–80% Belgium), and 40% stated that there were no such programs (range 0% Denmark–68% Spain and Portugal). Of the non-EU countries, 69% (Taiwan 46%, Israel 50%, and USA 74%) of the care providers reported that DSME is accessible to low-literate patient groups, while 15% said that there are no such programs (58% of the affected people: “don't know”). The experts who completed the survey indicated that approximately 50–60% of programs in the EU MS targeted at different age groups of individuals with DM take the patients' health literacy into account, while in other selected countries, 70–80% programs targeted at children, adults, and elderly people with diabetes are organized according to cultural and socioeconomic differences.

### 3.5. Knowledge for Managing Diabetes

**Figure 2:**
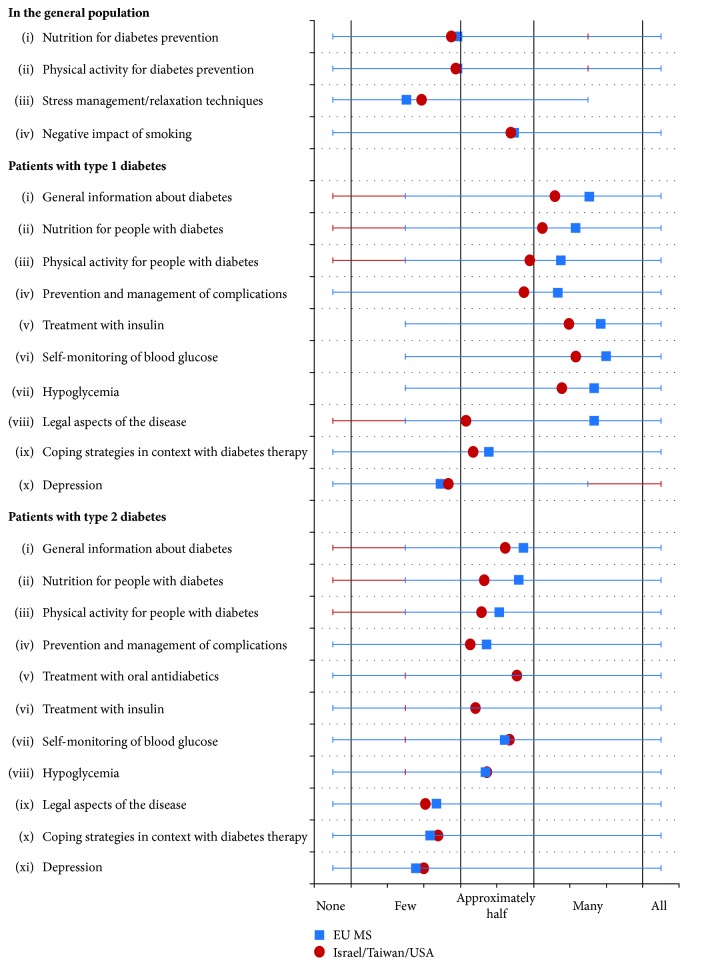
Estimated proportion of people having adequate knowledge of diabetes managing illness in everyday life (*n*/all EU MS = 249, *n*/Israel/Taiwan/USA = 130).

Regarding the disease management knowledge, the respondents of EU MS estimated that approximately half of the general population has sufficient knowledge about the negative impact of smoking (55%), one-fourth (25%) has adequate knowledge about stress management and relaxation techniques, and one-third (39% each) has adequate knowledge concerning physical activity for diabetes prevention and nutrition for diabetes prevention (Figure 1). Over half of the patients with T2DM were estimated to have sufficient knowledge to manage their illness on general information about diabetes (57%), nutrition for people with diabetes (56%), physical activity for people with diabetes (51%), prevention and management of complications (47%), treatment with oral antidiabetics or insulin (57% versus 44%), self-monitoring of blood glucose (52%), and hypoglycemia (47%). Knowledge was estimated to be insufficient with respect to legal aspects of the disease (34%), coping strategies in the context of diabetes therapy (32%), and depression (28%). However, patients with T1DM were estimated to have better knowledge on managing their illness (75%) than people with T2DM, except with regard to coping strategies in the context of diabetes therapy (48%) and depression (35%); the indicated values for all other categories were between 67% and 80%. There were minimal differences in the responses between different stakeholder groups and between the EU MS compared to the selected non-EU countries (ITU). The variation was especially large in the case of legal aspects (i.e., driver's license, traffic law, and labor law) of diabetes for T1DM patients (Figure 2).

Eighty-two percent (82%) of respondents stated that DSME programs promote patient empowerment and improve self-management. About the same proportion (83%) stated that they promoted a healthy lifestyle for people with diabetes (n.s. between different stakeholders in the EU MS and in ITU).

### 3.6. Participation in DSME

**Figure 3:**
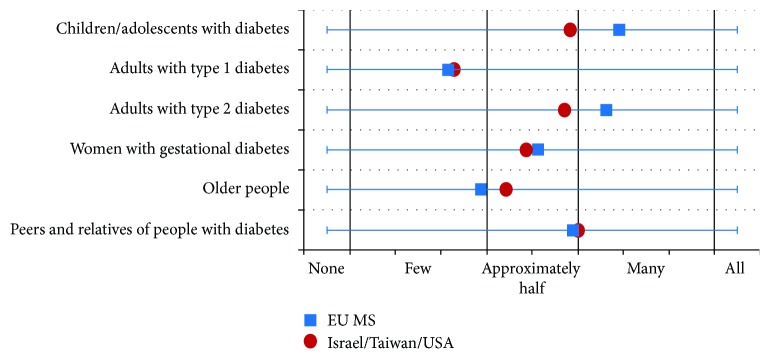
Participation of patient populations in DSME programs in EU MS and Israel, Taiwan, and USA.

According to the respondents of the EU MS and ITU, the proportion of adults with T1DM and older people that had participated in DSME programs was lower compared to that with other patient groups. On the contrary, in the EU MS and ITU, the estimated proportions of children/adolescents with diabetes and adults with T2DM participating in DSME were higher, but in ITU, these values were somewhat lower than in the EU MS (Figure 3). There were no significant differences between the answers of different stakeholder groups.

Regarding the existence of specific rules with respect to when a patient can attend DSME program, an average of 25% of the total EU MS sample stated “yes, based on need assessment”; 18% with “yes, following a recent diagnosis”; 12% with “yes, on an ongoing basis”; and 11% with “yes, after disease progression (e.g., starting insulin therapy)”; 33% responded “no” or “I don't know” (affected people 36% versus care providers 24%). All answers between the affected people and care providers varied significantly in the EU MS (chi^2^ test: *p* ≤ 0.05). In the selected non-EU countries (ITU), the answers did not vary significantly between the affected people and care providers.

### 3.7. Financing of DSME

According to the experts from EU MS, costs for the DSME for all age groups of individuals with diabetes are primarily covered by the health insurers (50–60%) and partially by the patients (approximately 20%). A similar trend was also observed in Israel, Taiwan, and the USA. However, the information regarding assumption of the costs for DSME targeting peers and relatives of people with diabetes remains unknown to most of the experts (65.2%) from EU MS.

The differences in payment strategies for DSME of patients with different insurers or payers were also reported in the expert survey: the average score was 1.7 ± 2.2 for EU MS versus 1.0 ± 0.0 for Israel and Taiwan in a scale of 1 (no difference) to 10 (huge difference).

### 3.8. Differences in DSME Programs between Different Regions

Based on the expert survey, there are regional differences in the DSME across EU MS regarding the costs, payment strategies, certification/accreditation, and quality assurance of the DSME programs. The average score for EU MS was 4.3 ± 2.9 (versus 2.7 ± 2.2 for Israel/Taiwan), with scale ranges 1 (no difference) to 10 (huge difference).

### 3.9. Status of DSME

On a scale ranging from 1 (no priority) to 10 (highly prioritized), respondents to the DLS rated the priority of diabetes self-management education at national level on average at 6.2 ± 1.4, with a range between 2.7 ± 0.6 to 9.0 ± 0.0. For the ITU, the rate was comparable. Affected people and other stakeholders estimated the priority as lower than the care providers. Regarding the satisfaction with the current state of DSME in the EU MS, the mean was 5.2 ± 1.4, on a scale of 10 (1 completely dissatisfied–10 extremely satisfied), with a range of 3.0 ± 0.0 to 9.0 ± 0.0. The respondents in ITU reported comparable rates (Israel: 4.7 ± 2.2; Taiwan: 5.5 ± 2.5; and USA: 4.8 ± 2.1). The estimates of the affected people and care providers both in the EU MS and ITU were comparable.

## 4. Discussion

This is the first study to assess the state of diabetes self-management education in the EU MS based on the views of multiple stakeholders. Results from EU MS were contrasted with those for Israel, Taiwan, and the USA in order to analyze and compare DSME in different health systems in countries that have a comparable Human Development Index score [19].

The results show a broad landscape of DSME programs in the EU MS and a great variety regarding the distribution: of the total of 102 DSME programs, nearly half were reported for one country (Germany; *n* = 54), while for several other countries, no programs were reported at all. The variation is partly due to different health care structures and responsibilities, but it could also be affected by language barriers in responding to the survey. This indicates that in order to complete the map of DSME programs in the EU MS, providing survey questionnaire for more languages (we used seven) and using further methods, such as systematic review, should be considered. The large number of identified programs also implies that the existing programs should be evaluated and modified to the needs of different patient groups and health systems across the EU MS.

Regarding the accessibility of the existing DSME programs to people with lower levels of literacy or lower educational/language attainment, our study revealed that approximately 29% of the respondents in the EU and 63% of the respondents from ITU considered the programs as accessible. This implies that the majority of the respondents (70%/EU MS, 61%/Taiwan, and 55%/Israel) were not aware of (“I don't know”/“there are no”) DSME programs tailored for people with lower levels of literacy or lower educational/language attainment, compared to 30% in the USA.

Health literacy impacts the management of diabetes in everyday life. Our survey results estimate that less than half of the general population in the EU has sufficient knowledge about the risk factors for the development of diabetes mellitus and subsequent complications: smoking, stress, and inadequate physical activities. People with T1DM were thought to have better knowledge about prevention and care of diabetes than people with T2DM, but the knowledge is considered inadequate regarding diabetes-related depression and coping skills. This suggests that the DSME strategies should include the components of diabetes prevention knowledge and methods for coping.

The reported data on participation of the affected people in the DSME programs implies that the potential of the DSME programs is underutilized in some of the EU MS. Especially for adults with T1DM and older people, they need to be motivated more to take part in DSME. Attending DSME correlates with organizational issues. In regard to the existence of specific rules stipulating a specified point in time to attend DSME program, the answers between the affected people and care providers varied significantly in the EU MS. Only a quarter of the respondents in the EU MS stated that the DSME program is based on needs assessment and only one-tenth on an ongoing basis. Nevertheless, these two methods should be valued as a standard to obtain the long-lasting effects of DSME. The results also imply that care providers should be more aware of the optimal point in time to conduct the DSME programs in order to inform and motivate their patients to participate in the DSME programs.

Affected people and other stakeholders estimated that diabetes self-management is of lower priority in the regional community compared to that estimated by the care providers in the EU MS and ITU. This suggests that the inadequate participation reported in various DSME programs could be the reflection of less prioritization of DSME in the community. The estimates between the affected people and care providers in the EU MS as well as in ITU were comparable regarding the satisfaction with the current state of DSME in the regional community, although there was a huge variation across the EU MS.

Results of the Diabetes Literacy Survey with multiple stakeholders and the expert survey suggest that the following aspects need more investment to improve DSME in the future:

- As many people with DM do not participate in the DSME programs even if such programs are free of charge, more research is needed to maximise the participation through flexible time schedule (e.g., late evening and weekend programs, IT/online based).
- Health care providers should empower patients to cope and manage DM. Thus, DSME should be integrated as participative element with emphasis on lifestyle changes, social and emotional support, and psychosocial adaptation of the patients and their family members—and not just as a passive delivery of health knowledge.
- In order to have sustained effect, the DSME should be tailored to the patient needs and continued throughout the life course. In addition, clinicians should have more time to motivate people to attend DSME.
- Governments/health insurers should provide financial support for unified structured DSME system to reduce social and health inequalities.
- Information regarding existing DSME programs should be made available for all stakeholders. Existing DLS reported programs can be found in http://dsme.wiki/. Reporting more programs on the website is possible on an ongoing basis.
- Policy-level education strategies are needed as part of the solution to overcome the diabetes epidemic in EU MS and beyond. To reach the population level effects, DSME national plans should be implemented in all EU MS, and the programs should be evaluated on a regular basis for their effectiveness and cost-effectiveness.

### 4.1. Implications of the Study

Our data related to the state of self-management education targeting people with diabetes, as well as care providers in the EU MS from multistakeholder perspective, will contribute to a currently insufficient evidence base to inform policy makers on improving the DSME to include prevention, treatment, and care for the diversity of patient groups with diabetes. The importance of this study lies in developing the necessary evidence that is needed to build a comprehensive diabetes strategy at EU level. This is a great challenge considering the heterogeneity of European health systems, national plans, and models of DSME; diversity of languages and cultures; and economic and political circumstances across the EU MS.

### 4.2. Limitations

The results of this study are based on self-reporting and are not objectively validated. Since participation rates were not comparable in all EU MS and were especially low in the Eastern European countries, this study did not provide fully comprehensive data coverage. The comparison between EU MS and non-EU states was based on a disproportionate number of participants regarding people with diabetes and care providers (more people with diabetes in EU MS than non-EU states and therefore more care providers in non-EU states). Therefore, it is not possible to claim the representativeness of the data.

Heterogeneous participation rates could be associated with the available seven languages of the survey questionnaire, implying that the future questionnaire should include more languages. Bias based on representativeness was avoided by analysing EU MS data in country-specific analysis only in the case where there was a minimum of 8 participants per each country.

The reported data are partly based on DSME knowledge currently offered in different health systems and programs which may change from year to year that limits comparing the state of DSME offered across the countries. Further, self-reported data may contain sources of bias such as recall bias. Verifying data with other sources was not possible, since this was the first study to collect views of multiple stakeholders on DSME.

## 5. Conclusions

In conclusion, variation of DSME in the EU MS, partly due to different health systems, is a great challenge for developing an EU-wide diabetes strategy. Our study demonstrates that people with low or limited health literacy skills [14] should be put in focus in designing DSME, since low health literacy skills strongly affect the ability for disease self-management and negatively correlate with health status. In addition, both older and younger people with diabetes need special attention regarding the design and organization of DSME programs across the EU MS. Furthermore, DSME is mainly provided by diabetes specialists, for example, diabetes educators, diabetes nurses, and general practitioners, in the EU MS and ITU, where the satisfaction with DSME programs was comparable. The role of specially trained diabetes nurses and other health professionals in DSME must be supported further, augmenting the work of general practitioners to impart the self-management skills necessary to control the disease and use health resources more efficiently.

## Figures and Tables

**Table 1:** Providers of diabetes self-management education (incl. educators and frameworks) in the EU MS and Israel, Taiwan, and the USA (all respondents based on the Diabetes Literacy Survey).

Providers of DSME in the EU MS, Israel, Taiwan, and the USA (ITU)	EU MS	ITU
Diabetes specialist (diabetologist/endocrinologist)	60%	61%
General practitioner	41%	54%
Diabetes/health educator	36%	72%
Diabetes nurse	41%	58%
Primary care nurse	19%	19%
Diabetes education team in a hospital or a rehabilitation clinic	37%	44%
Pharmacists	10%	18%
Health insurance providers	5%	18%
Other health-related organizations	4%	13%
Diabetes support group/diabetes organization	36%	40%
Patient to patient (e.g., peers or lay health workers)	10%	16%
Patient self-directed learning (e.g., IT platforms or journals)	20%	36%
Other	6%	11%
Don't know	2%	2%

**Table 2:** For which target groups do structured diabetes (self-management) education programs exist in your regional community? Percentage of all respondents answering “yes” based on the Diabetes Literacy Survey.

	Children/adolescents with diabetes	Adults with type 1 diabetes	Adults with type 2 diabetes	Women with gestational diabetes	Older people	Peers and relatives of people with diabetes	Others
EU total (*n* = 249)	61	73	73	47	42	33	4
Selected EU MS (*n* = 197)	61	74	74	46	46	35	5
Austria (*n* = 28)	57	68	86	39	54	29	0
Belgium (*n* = 10)	80	90	80	60	40	20	0
Denmark (*n* = 8)	75	50	63	50	25	38	0
Germany (*n* = 70)	66	84	80	50	60	44	10
Ireland (*n* = 13)	38	77	92	31	15	31	0
Portugal (*n* = 30)	59	59	55	48	38	34	0
Romania (*n* = 9)	78	78	56	33	44	22	0
Spain (*n* = 10)	90	60	50	70	40	40	0
UK (*n* = 19)	37	79	79	32	37	21	11
Other EU MS (*n* = 52)	60	69	71	52	27	25	4
Other total (*n* = 130)	60	68	85	61	62	44	8
Israel (*n* = 9)	67	56	78	56	67	22	11
Taiwan (*n* = 26)	54	38	58	35	50	42	0
USA (*n* = 95)	61	77	93	68	65	46	9

**Table 3:** Are there diabetes (self-management) education programs designed to be accessible for people with lower levels of literacy, lower educational attainment, or lower language attainment? Percentage of all respondents based on the Diabetes Literacy Survey.

	Yes	No	Don't know
EU total (*n* = 249)	29	35	35
Selected EU MS (*n* = 197)	29	35	35
Austria (*n* = 28)	22	10	10
Belgium (*n* = 10)	40	40	20
Denmark (*n* = 8)	50	13	38
Germany (*n* = 70)	25	33	40
Ireland (*n* = 13)	46	38	15
Portugal (*n* = 30)	21	38	41
Romania (*n* = 9)	44	44	11
Spain (*n* = 10)	30	30	40
UK (*n* = 19)	37	32	32
Other EU MS (*n* = 52)	27	37	37
Other total (*n* = 130)	63	15	23
Israel	44	22	33
Taiwan	39	23	38
USA	70	12	18
